# ECG Changes Occurring During Chest Liposuction for Gynecomastia – Artifacts or a Cause for Concern?

**DOI:** 10.7759/cureus.32850

**Published:** 2022-12-22

**Authors:** Ved Prakash Rao Cheruvu, Sri Rama Ananta Nagabhushanam Padala, Manal M Khan, Pragna Reddy Basireddy

**Affiliations:** 1 Plastic and Reconstructive Surgery, All India Institute of Medical Sciences, Bhopal, IND; 2 Anesthesiology and Critical Care, All India Institute of Medical Sciences, Bhopal, IND

**Keywords:** adult, male, nipples, liposuction, gynecomastia

## Abstract

Currently, liposuction alone or combined with various methods of gland excision has become the standard of care in the surgical management of gynecomastia. Although liposuction is considered a safe and straightforward method of body contouring, serious complications related to the procedure, including deaths, have been reported in the literature. We report the occurrence of ECG artifacts intra-operatively while performing chest liposuction under general anesthesia. Patients may receive unnecessary and potentially dangerous therapeutic interventions if these ECG artifacts are not identified correctly. A careful and rational evaluation of the patient and the ECG by the clinician can accurately identify these pseudo-abnormalities and avoid unnecessary therapies.

## Introduction

“Gynecomastia” refers to the enlargement of the male breast due to the proliferation of ductal, stromal, or fatty tissue. It commonly affects up to 65% of men [1]. Patients with aesthetic concerns seek surgical correction. The advent of liposuction and the further development of its variants, like power-assisted and ultrasound-assisted liposuction, have made the surgical treatment of gynecomastia less invasive [1]. Currently, liposuction alone or in combination with various methods of gland excision has become the standard of care in the surgical management of gynecomastia [2].

Liposuction is the second most commonly performed cosmetic surgery in the United States and the most common surgical procedure in patients between the ages of 35 and 64 years [3]. The number of liposuction procedures performed increased by 124% from 1997 to 2015 [4]. Although liposuction is considered a safe, simple, and effective method of body contouring [5], an overall complication rate ranging from 0 to 10% and a mortality rate of one death in 5000 procedures performed has been reported [6]. The reported complications can be correlated to the physician’s expertise and experience, technical deficiencies, aseptic precautions, tumescent anesthesia, fluid overload, large volume liposuction, performing multiple procedures in one setting, embolism, and inadequate postoperative monitoring [6]. Liposuction often is performed as an outpatient daycare procedure [7].

Jeffrey Klein, a pioneer of tumescent liposuction, pointed out the risks associated with general anesthesia when performing liposuction. In his view, virtually all liposuction-related deaths had been associated with using general anesthesia or heavy intravenous sedation [6]. Therefore, to avoid serious complications, careful intra-operative monitoring is required for liposuction procedures performed under general anesthesia or intravenous sedation.

## Technical report

**Figure 1:**
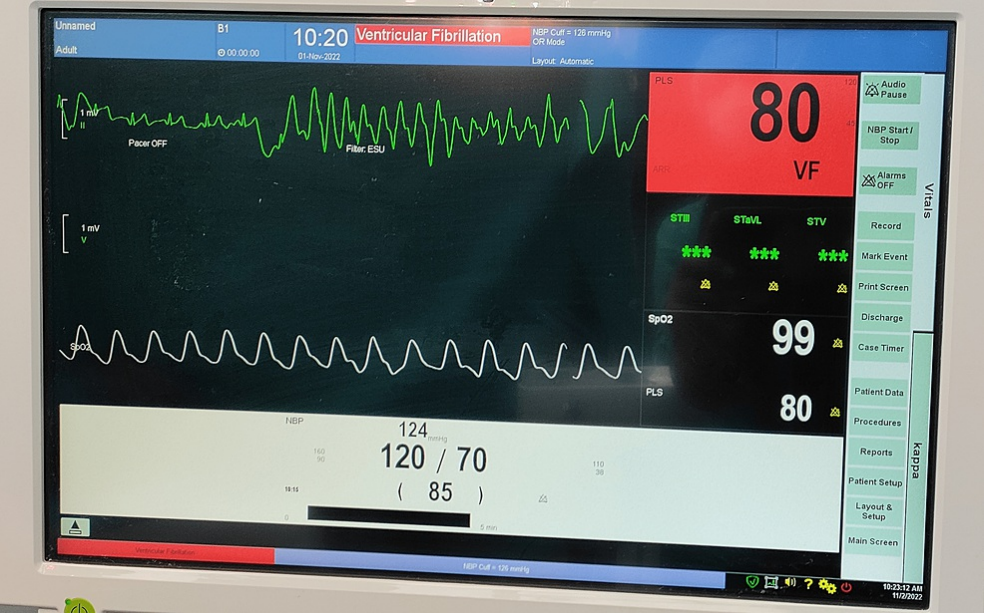
ECG changes occurring during chest liposuction in a 30-year-old healthy male with no pre-existing cardiac disease

**Figure 2:**
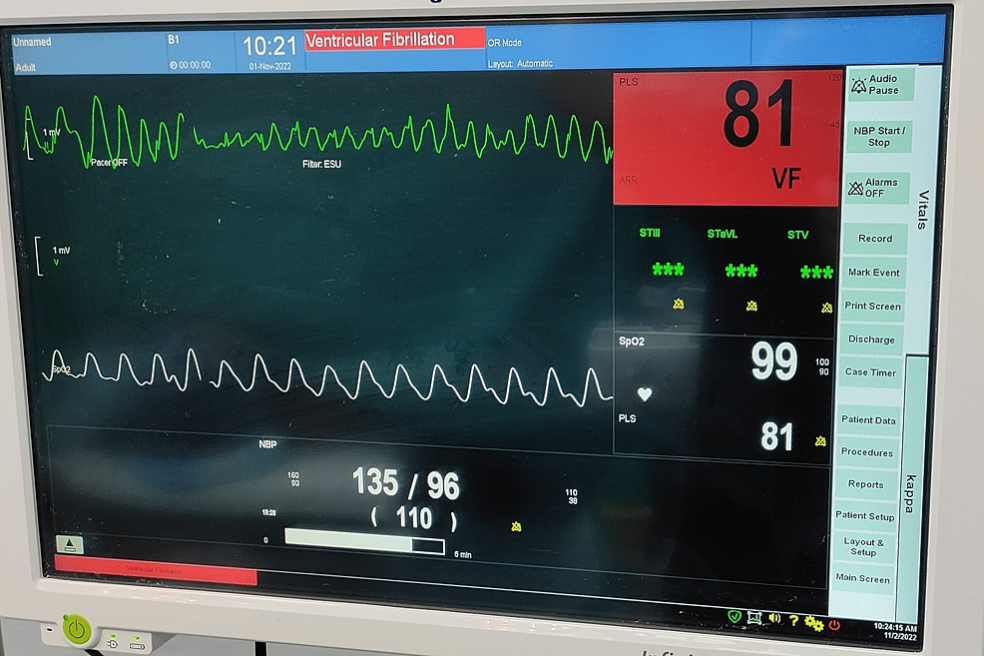
ECG changes occurring during chest liposuction in a 30-year-old healthy male with no pre-existing cardiac disease

**Figure 3:**
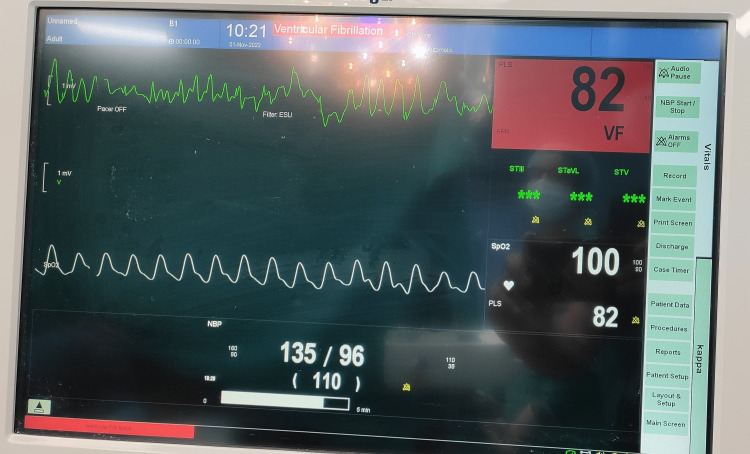
ECG changes occurring during chest liposuction in a 30-year-old healthy male with no pre-existing cardiac disease

We report the occurrence of electrocardiogram (ECG) abnormalities intra-operatively while performing chest liposuction under general anesthesia. They can occur as long as the stroking action of the liposuction cannula is performed and resolve on stopping. Figures 1-3 demonstrate the ECG aberrations seen while chest liposuction was performed under general anesthesia in a 30-year-old healthy male with no pre-existing cardiac disease.

Except for the artifacts in ECG during liposuction, the pulse oximetry tracing and blood pressure can be seen to be normal. The procedure and the recovery were uneventful.

**Figure 4:**
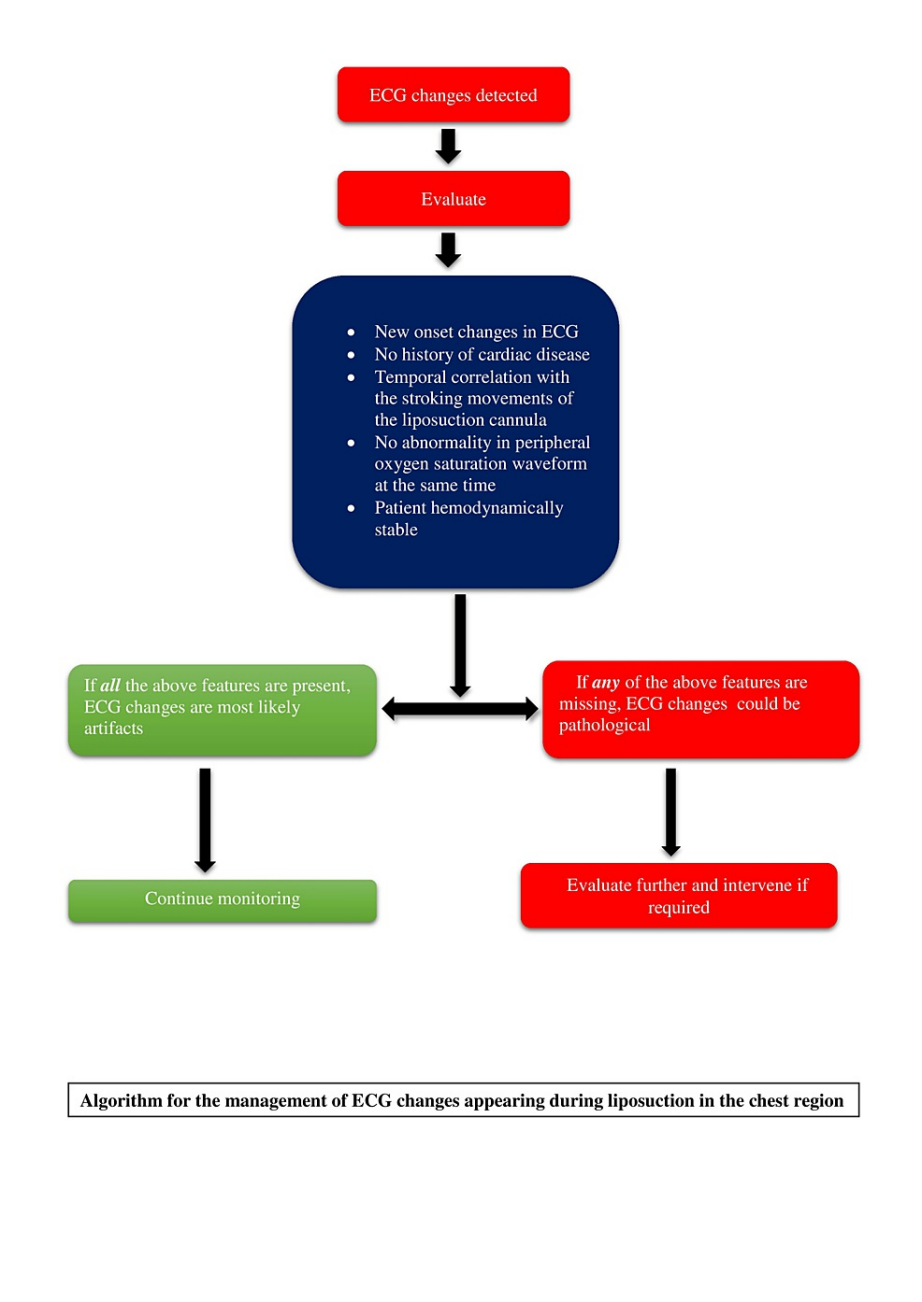
Algorithm for the management of ECG changes appearing during liposuction in the chest region

Any ECG abnormality unrelated to the cardiac electrical activity can be termed an artifact [8]. These ECG artifacts can mimic abnormalities like ventricular tachycardia, atrial flutter, pacemaker rhythm, and non-specific ST-T changes. These most probably are motion artifacts caused by epidermal stretch-induced voltage change. This effect originates from the ECG lead contact with the skin. The ECG has demonstrated that a voltage difference of several millivolts is recorded simply by stretching the epidermis. Artifacts may mimic the range of pathological electrocardiographic findings, ranging from arrhythmia to ischemic change. If these ECG artifacts are not identified correctly, patients may receive unnecessary and potentially dangerous therapeutic interventions [8]. A careful and rational evaluation of the patient and the ECG by the clinician can accurately identify these pseudo-abnormalities. A clinical correlation between a stable patient and a malignant ECG abnormality should arouse the suspicion of an artifact. We suggest an algorithm for evaluating ECG changes appearing during liposuction in the chest region (Figure 4).

## Discussion

Although liposuction is considered a relatively safe procedure, complications can occur. The ECG artifacts during chest liposuction need to be differentiated from the ECG abnormalities, which occur due to pathological causes like arrhythmias due to tumescent solution, fluid overload, and pulmonary causes like pulmonary edema, ARDS, and fat embolism syndrome. The absorption of the tumescent solution from the subcutaneous tissues to the intravascular space can lead to pulmonary edema. Administration of high doses of lignocaine during tumescent anesthesia can result in toxicity, leading to cardiac conduction and contraction abnormalities and fatal arrhythmias. Adrenaline in the wetting solution may also produce arrhythmias if the circulating levels are high. Hypothermia, especially in patients undergoing large-volume liposuction, may worsen cardiac dysrhythmias.

The various causes of ECG artifacts have been described [8]. The differentiation between artifactual ECG changes and cardiac rhythm abnormalities is vital for patient safety. In a patient who is otherwise asymptomatic and hemodynamically stable, it is important to consider the possibility of an artifact while evaluating the ECG changes. The temporal correlation of the stroking action of the suction cannula during chest liposuction to the appearance of ECG changes also supports the diagnosis of an artifact. Similar ECG artifacts can occur during shivering, shaking during delirium, and Parkinsonian tremors [9-11]. A vigilant and thoughtful clinician can differentiate between artifacts and pathological ECG changes and avoid administering unnecessary and possibly dangerous therapies.

## Conclusions

Identifying motion artifacts in ECG while performing chest liposuction for gynecomastia can avoid inappropriate pharmacological interventions. We need a careful and rational evaluation of the patient and the ECG pattern to differentiate artifacts from pathological ECG changes.
